# Measurement of electrocardiograms in a bath through tap water utilizing capacitive coupling electrodes placed outside the bathtub wall

**DOI:** 10.1186/s12938-016-0304-9

**Published:** 2017-01-11

**Authors:** Kosuke Motoi, Yasuhiro Yamakoshi, Takehiro Yamakoshi, Hiroaki Sakai, Naoto Tanaka, Ken-ichi Yamakoshi

**Affiliations:** 1 Shizuoka Institute of Science and Technology, 2200-2 Toyosawa, Fukuroi, 437-8555 Japan; 2 Hokkaido University, Kita 14, Nishi 9, Kita-ku, Sapporo, 060-0814 Japan; 3 Fukuoka Institute of Technology, 3-30-1 Wajiro-higashi, Higashi-ku, Fukuoka-shi, Fukuoka, 811-0295 Japan; 4 Spinal Injuries Center, 550-4 Igisu, Izuka, Fukuoka, 820-8508 Japan; 5 NPO Research Institute of Life Benefit, 6-11-7-1 Sumikawa, Minami-ku, Sapporo, 005-0006 Japan; 6 Showa University School of Medicine, 1-5-8 Hatanodai, Shinagawa-ku, Tokyo, 142-8555 Japan; 7 Kanazawa University, Kakuma-machi, Kanazawa, 920-1192 Japan

**Keywords:** Non-invasive and non-intrusive health monitoring, Home facilities, Bathing, Bathtub wall, Capacitive coupling electrodes, Electrocardiogram (ECG), Respiration, RR interval

## Abstract

**Background:**

Taking a bath sometimes poses a risk for subjects with chronic cardiopulmonary disorders, due to the thermal effect and water pressure on his/her body. The ECG measurement would be helpful for the early recognition of abnormal cardiac beats and respiratory conditions. This paper describes a new attempt to improve on previous bathtub ECG measurement techniques that had electrodes placed inside the bathtub that were intrusive to the subjects’ bathing experience. This study is concerned with the initial development of a method to measure an electrocardiogram (ECG) through tap water without conscious awareness of the presence of electrodes that are placed outside the bathtub wall.

**Methods:**

A configuration of capacitive coupling electrodes placed outside the bathtub was designed so that the electrodes could be hidden. The capacitive coupling was made from the electrodes to the water through the bathtub wall. Two electrodes with an active shielding amplifier covered further by an electromagnetic shield were fixed to the outside surface of the bathtub wall, near the bather’s right scapula and left foot. The potential difference between these two electrodes, similar to the bipolar lead-II ECG, was amplified to obtain raw signals inclusive of ECG/QRS components. Respiration intervals were also derived from ECG/RR intervals. Comparison experiments between this bathtub method and conventional direct methods with spot-electrodes and a chest-band sensor were made using 10 healthy male volunteers (22.2 ± 0.98 years).

**Results:**

The ECG signal was detectable through tap water as well as water with differing conductivity resulting from mixing bathwater additives with the water. ECG signals and respiration curves derived from ECG/RR intervals were successfully obtained in all subjects. The intervals of the ECG/RR and respiration obtained by the bathtub system and by the direct method were respectively agreed well with each other.

**Conclusion:**

The ECG signal, in particular ECG/QRS components, were successfully detected utilizing capacitive coupling electrodes placed outside the bathtub wall. Also, the ECG/RR and respiration intervals were determined with reasonable accuracy as compared with the conventional direct methods.

## Background

Daily monitoring of health conditions at home has been well recognized as important for the prevention of lifestyle-related chronic diseases such as adiposis, diabetes, cardiopulmonary disease and others. Commercially available devices, such as an automated sphygmomanometer or a set of scales, are widely used for health monitoring at home, with more recent devices also having data transmission functions using Bluetooth, Wi-Fi or other networking technologies. Although such health monitoring appears easy and convenient, the continuous use of such devices becomes cumbersome for the subject, because of the need to attach biological sensors to his/her body as well as to operate the devices. These cumbersome procedures lower the users’ motivation to continue monitoring for a period of time that is long enough to check their health condition.

With the above mentioned points in mind, this paper proposes a new concept of non-invasive health monitoring to measure physiological variables without interrupting normal daily life activities in a fully automated manner without the need for the subject to attach any biological sensors to their body or operate complicated measurement devices [1–3]. In non-invasive health monitoring, all of the necessary sensors are built into existing home facilities such as the toilet, bathtub, and bed. Many developments have been made to implement non-invasive health monitoring. For example, a device that can measure the electrocardiogram (ECG) [4], temperature [5], cardiac pulse, respiration and body motions during sleep [6–12] has been installed into a bed. Additionally, recent progress in information and communication technologies has made it possible to measure pulse rate and pulse volume using only a smartphone [13, 14].

While taking a bath, it is well known that there can be a danger of accidents such as a sudden malfunction of cardiopulmonary systems following thermal and water pressure load to the body [15]. Cardiopulmonary monitoring during bathing in a subject to be cared for at home, especially with chronic cardiopulmonary disorder, would therefore be helpful for early detection of abnormal cardiac beats and respiratory conditions as well as for prevention of drowning caused by a sudden heart attack [3, 15]. Taking these circumstances into account, a system for ECG and/or respiration measurement during bathing has been developed using bathtub-installed electrodes [16–18]. Even if abnormal rhythms of cardiac beatings as well as respiration could not be observed by medical examinations in a hospital, the bathtub method has potential to catch an abnormal symptom during bathing [18, 19].

However, the previous bathtub methods featured electrodes placed inside the bathtub wall, and thus a subject could see the electrode directly. Such visible electrode could usually provide a feeling of strangeness and/or discomfort for the bathing subject. In fact, it has been reported from medical evaluations [18, 20], that the patients worried about the presence of the electrodes and thus visible electrodes were considered undesirable. Furthermore, such electrodes corroded over time due to repeated direct contact with water in the bathtub and thus the ability to record the ECG signal gradually declined.

On the other hand, a “capacitive coupling electrode” technique, that does not require the use of body surface electrodes, has been designed to measure ECG signals [21–26]. This technique also does not need moisture such as an electrical conductive gel between the electrode and the skin and it is possible to acquire the ECG signal through the subject’s clothing.

The present study is an attempt to apply this technique to ECG measurement through the bathtub wall: A configuration of capacitive coupling electrodes placed outside the bathtub wall was designed so that the electrodes could be hidden. In this study, the measurement of ECG/QRS components was in particular concerned with possible detection of ECG/RR intervals. The performance of this bathtub system was described through comparison experiments between the values of ECG/RR intervals determined by the bathtub method and by a conventional direct method with spot-electrodes. The respiration intervals by the derivation of the bathtub ECG/RR intervals and a direct method with a chest-band sensor attached to the body surface were also compared.

## Methods

### Description of bathtub ECG measurement system

**Fig. 1:**
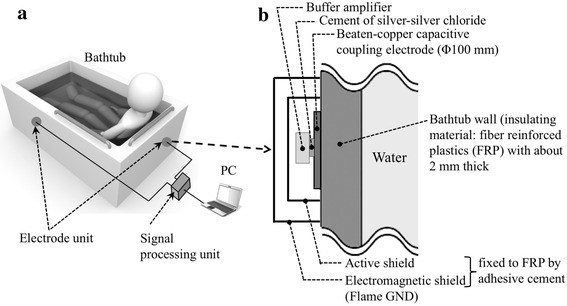
Outline of bathtub electrocardiogram (ECG) measurement system using capacitive coupling electrodes through the bathtub wall. **a** Shows electrode arrangement and **b** structural drawing of the electrode-unit

Figure 1 shows an outline of the bathtub ECG measurement system, consisting of two electrode-units with a buffer amplifier, a signal processing unit and a conventional PC. The capacitive coupling was made between the 100 mm diameter electrodes placed outside the bathtub wall and the water. A commercial bathtub (SP1272, NORITZ) made of fiber reinforced plastics (FRP; electric permittivity of this insulating material is about 4.5) was used for this study. After surface polishing of about 2 mm thick of the bathtub wall, electrically conductive coating (DOTITE, Fujikura Kasei Corp., Tokyo, Japan) was applied to the wall to produce the beaten-copper electrode. This copper electrode and the terminal (10 × 10 mm^2^) of the buffer amplifier were bonded with each other using an adhesive cement. If the impedance of this cement becomes high with time, the signal may be decreased. The adhesive cement including silver–silver chloride (AgAgCl) materials (011464, BAS Inc., Tokyo, Japan) was therefore used to maintain a low contact resistance for a long period of time. This active electrode was covered by an active shield and further by an electromagnetic shield (see Fig. 1b) to maintain the uniformity of the magnetic field around the electrode. The two electrode units thus constructed were fixed to the rear surface of the bathtub wall using an adhesive cement, near the subjects’ right scapula and left foot (see also Fig. 1a).

**Fig. 2:**
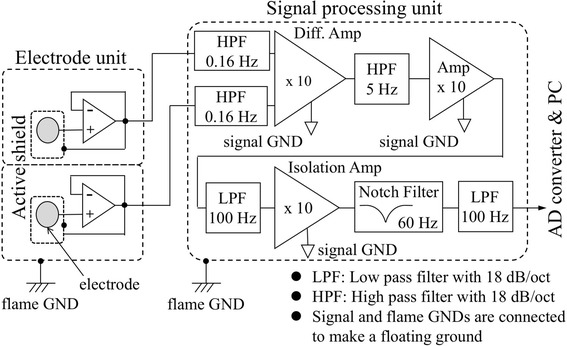
Block diagram of the electrode-unit and the signal processing unit to obtain bathtub ECG signals

Figure 2 is a block diagram of the electrode-unit and the signal processing unit designed by us, which is composed of low-pass (LPF) and high-pass filters (HPF) and amplifiers. The signals from each buffer amplifier (OPA277, Burr Brown Corp., Tucson, USA) was sent to the HPF (0.16 Hz cut-off frequency) and then to a differential amplifier: Diff. Amp (INA128, Texas Instruments Inc., Dallas, USA). The output from this amplifier, the potential difference between the two electrodes corresponding to a bipolar lead-II ECG, was taken as a raw ECG signal.

To acquire stable ECG signals including QRS components and respiratory intervals derived from ECG/RR intervals, low frequency components such as respiratory fluctuations and artifacts caused by slight body movements in the bathtub were reduced by adopting the 5 Hz high pass filter. This signal was further processed through two LPFs (100 Hz), a notch filter (SD-1BE, NF Corp., Yokohama, Japan) to decrease the power-supply noise, and an amplifiers (OPA2277, Burr Brown Corp., Tucson, USA) and an isolation amplifier (AD204KY, Analog Devices Inc., Norwood, USA) with total gain including Diff. Amp of 10 × 10 × 10 (=1000).

The electrical grounding (GND) was not connected to the earth. The signal and flame GNDs are thus floating GND. The tap water inside the bathtub was also not connected to the earth. While there was electrical noise from the surrounding environment, all signal levels including the GND fluctuated in a similar way, and thus the noise could be cancelled, obtaining a practically acceptable common mode rejection ratio (about 60 dB). Therefore, a third electrode with driven right leg [27] was not used in this study.

The signal was finally led to the PC via an AD converter (16 bits, 300 Hz of sampling frequency and voltage range of ±5 V; NI USB-6218, National Instruments Corp., Austin, USA). In this way, it was possible to obtain ECG/RR and respiratory intervals as derived from ECG/RR intervals based on respiratory sinus arrhythmia [28] using an automated analysis program (MATLAB, MathWorks).

### Algorithm

**Fig. 3:**
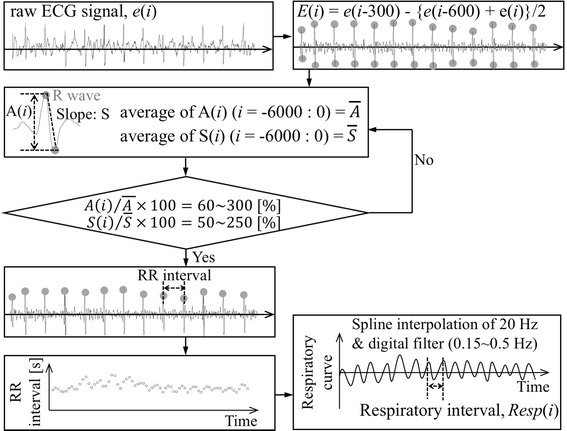
Flowchart algorithm for the determination of ECG/RR and respiration intervals from ECG signals

Figure 3 shows a flowchart algorithm for the determination of beat-by-beat ECG/RR (RR interval in this Fig. 3) and respiration interval [*Resp*(*i*)] from the raw ECG signal [*e*(*i*)]. To emphasize the R waves, the *e*(*i*) is transformed into an *E*(*i*) signal according to the following equation.

$$E\left( i \right) \, = e\left( {i - 300} \right) - \left\{ {e\left( {i - 600} \right) + e\left( i \right)} \right\}/ 2$$

*i*: current data point.

The peak to peak value in the *E*(*i*) signal, *A*(*i*), and the slope, *S*(*i*), just after the R waves are then calculated. The average values of *A*(*i*), $\bar{A}$, and *S*(*i*), $\bar{S}$, in the past 6000 data points (20 s) are also obtained. If the percentage of $A\left( i \right)/\bar{A}$ is 60~300% and that of $S\left( i \right)/\bar{S}$ is 50~250%, *E*(*i*) is determined to contain R waves. Hence, the RR intervals (s) can be obtained from the R waves in the *E(i)*.

Additionally, RR intervals contain fluctuations synchronized with respiration due to respiratory sinus arrhythmia [28], and thus the *Resp*(*i*) can also be obtained from the RR interval data. From time series data of RR intervals (see the lowest left part of Fig. 3), a spline interpolation of 20 Hz and a digital filter of 0.15~0.5 Hz are processed to obtain a respiratory fluctuation curve, and therefore *Resp*(*i*) can be determined as the interval between the positive peaks.

### Comparison experiments and participants

A total of 11 healthy volunteers (all male; 22.2 ± 0.94 years) participated in the study. Firstly, we made a bathtub ECG measurement with two different conductivity water in the bathtub using one of 11 subjects randomly selected; one was with tap water whose conductivity was about 21 mS/m, and the other with conductivity water mixing a bathwater additive with conductivity of about 46 mS/m popularly used at home. In this experiment, the subject did not attach any electrodes and sensors to his body. As the first feasibility study in a healthy male subject (22 years), we evaluated whether the ECG signal can be obtained without the common spot-electrode used in the conventional ECG measurement. The bathtub ECG signals were measured in the tap water (the conductivity was about 21 mS/m).

Secondly, comparison experiments between the bathtub method and conventional direct methods were made using the remaining 10 subjects (22.2 ± 0.98 years) in the tap water. Based on the algorithm program mentioned above, the automatically determined values of the bathtub ECG/RR intervals and the corresponding ECG/RR intervals obtained by a conventional direct method with spot-electrodes attached to the body surface (lead-II) were compared simultaneously. The spot-electrodes were covered with a waterproof film (Tegaderm Film, 1629, 3 M, Maplewood) and thus never directly contact to the water. While, the values of respiration intervals obtained by the bathtub ECG/RR intervals and by a conventional direct method with a chest-band sensor (TR-753T, Nihon Kohden Corp., Tokyo, Japan) were also compared simultaneously. The chest-band sensor was also covered with the waterproof film. In this experiment, the tap water without the bathwater additive was used. It should be noted that all of the values used in this study were automatic determined without any manual data selections.

The participants were asked to take a bath in a usual fashion without body motions as far as possible for 420 s and to control their respiration conditions of rest (0~300 s), deep breath (300~360 s), simulated drowning (360~390 s), and rest once again (390~420 s). The beat-by-beat ECG/RR intervals were continuously obtained during the period. The breath-by-breath respiratory intervals were also detected during the rest and deep breath phases.

## Results

### Measurement of ECG signals

**Fig. 4:**
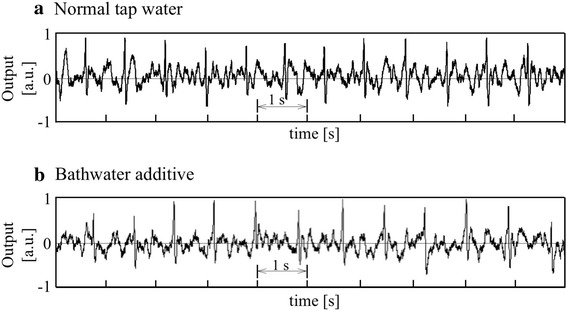
Example recordings of bathtub ECG signals obtained from tap water (approximately 21 mS/m of conductivity) (**a**) and conductivity water with the bathwater additive (approximately 46 mS/m) (**b**) on condition without the body-attached common electrodes in a subject (22 years). The *vertical axis* in each record is shown as arbitrary unit (a.u.)

Figure 4 shows example recordings of ECG signals obtained from tap water (a) and conductivity water with the bathwater additive (b), indicating that the ECG signals could be obtained without the use of conventional common electrodes inside the bathtub wall and that they were less influenced by water conductivity in the bathtub.

**Fig. 5:**
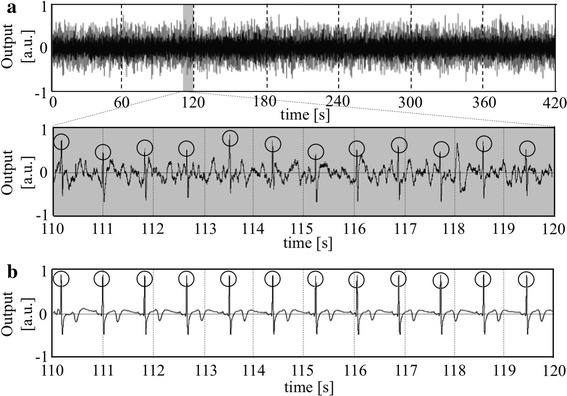
The *upper panel* is an example of a typical ECG record obtained from a different subject of Fig. 4 (22 years). The* middle panel* **a** shows an expanded record in time from 110 to 120 s in the *upper* record, and the *lowest panel* **b** is its corresponding record of **a** simultaneously measured by the direct method using spot-electrodes. *Circular marks* in each **a** and **b** indicate the ECG-R waves. The *vertical axis* in each record is shown as arbitrary unit (a.u.)

In the upper panel of Fig. 5 is shown a typical ECG record obtained from a subject (22 years). The middle panel (a) shows an expanded record in time from *t* = 110 s to *t* = 120 s in the upper record, and the lowest panel (b) is its corresponding record of (a) simultaneously measured by the direct method using spot-electrodes. Circular marks in each (a) and (b) indicate the ECG/R waves, clearly demonstrating a synchronization of both ECG signals, though noise components superimposed on the bathtub ECG signals were observed. However, QRS components of the bathtub ECG signals were clearly detected.

**Fig. 6:**
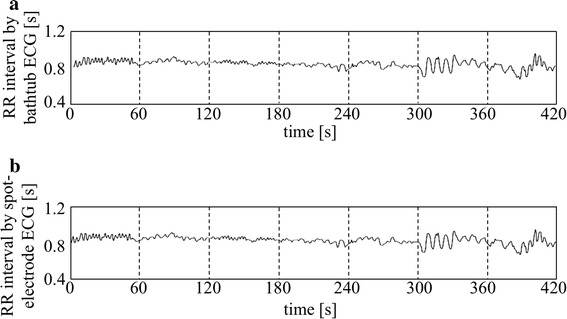
Trend charts of time-series data of ECG/RR intervals obtained by the bathtub method (**a**) and by the direct method (**b**) in the same subject of Fig. 5

Figure 6 shows trend charts of time-series data of ECG/RR intervals obtained by the bathtub method (a) and by the direct method (b) in the same subject of Fig. 5 during the experimental time. It is demonstrated that the changes in RR intervals coincide well with each other.

**Fig. 7:**
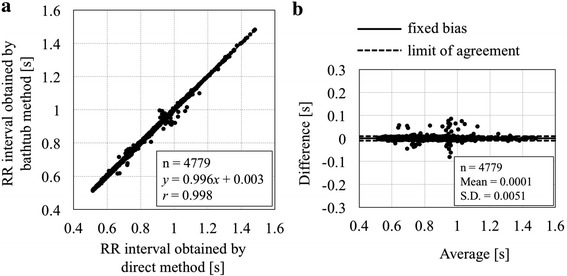
A scatter diagram between RR intervals obtained by the bathtub method and by the direct method in 10 healthy subjects (**a**), together with *Bland*–*Altman* plots (**b**). See text for further explanation

Figure 7a shows a scatter diagram between RR intervals obtained by the bathtub method and by the direct method in 10 healthy subjects. Altogether 4779 paired data sets were analyzed as follows: the linear regression line was *y* (bathtub method) = 0.996*x* (direct method) + 0.003 with correlation coefficient *r* = 0.998. *Bland*–*Altman* plots are also shown in Fig. 7b. The fixed bias was +0.0001 s and the limit of agreement was from −0.0099 to 0.0101 s.

### Respiration measurement

**Fig. 8:**
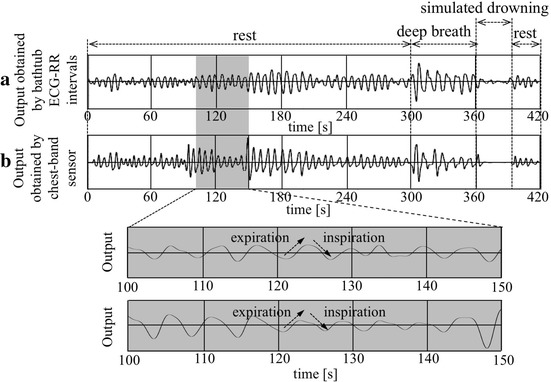
A typical example of simultaneously measured respiration curves obtained from the bathtub method (**a**) and from the direct method using the chest-band (**b**) during bathing in a healthy subject. The records of three conditions during periods of rest (0~300 and 390~420 s), deep breath (300~360 s) and simulated drowning (360~390 s) are shown in this example. The *lower two panels* show an expanded record in time from 100 to 150 s from the corresponding upper two records

Figure 8 shows a typical example of simultaneously measured respiration curves obtained from the bathtub method (a) and from the direct method using the chest-band (b) during bathing in a healthy subject. The records of three conditions during periods of rest (0~300 and 390~420 s), deep breath (300~360 s) and simulated drowning (360~390 s) are shown in this example. The lower two panels show an expanded record in time between *t* = 100 s and *t* = 150 s from the corresponding upper two records, indicating that the exhalation and inhalation phases (indicated by arrows) correspond well with each other. It is also noted that the decrease in amplitude of respiration curves during drowning can be detected.

**Fig. 9:**
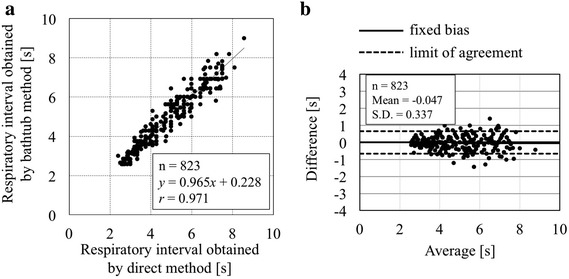
A scatter diagram between respiratory intervals obtained by the bathtub method and by the direct method in 10 healthy subjects (**a**), together with *Bland*–*Altman* plots (**b**). For further explanation see text

Figure 9a shows a scatter diagram between respiratory interval obtained by the bathtub method and by the direct method in 10 healthy subjects. Altogether 823 paired data sets were analyzed as follows: The linear regression line was *y* (bathtub method) = 0.965*x* (direct method) + 0.228 with correlation coefficient *r* = 0.971. Figure 9b shows *Bland*–*Altman* plots. The fixed bias was −0.047 bpm and the limit of agreement was from −0.708 to 0.613 s.

## Discussion

Through the present study, it is clearly demonstrated that ECG signals inclusive of QRS components can be successfully detected using the capacitive coupling electrodes attached to the outer side of a bathtub wall made of FRP which is widely used for commercial bathtub’s material. The conductivity change of bathtub water didn’t largely influence the ECG measurement as shown in Fig. 4. Although some noises were observed in the bathtub ECG signals compared to the ECG signals measured from body surface electrodes (see Fig. 5), appropriately processed ECG/RR intervals could nevertheless be accurately obtained, as demonstrated in Fig. 7. Some errors were noticeable which can be attributed to mistakes in the automatic detection of R-waves. Hence, further improvement of its software will be needed to increase accuracy.

Additionally, the respiration curve processed with the spline interpolation and digital filter from the time-series data of the bathtub ECG/RR intervals was similar to that measured by the direct method using the chest-band sensor (see Fig. 8). Therefore, it can be concluded that respiratory intervals are measured with reasonable accuracy using the bathtub-installed system (see also Fig. 9).

Taking these experimental findings into consideration, this bathtub ECG measurement technique has potential applications for hospital inpatients and outpatients especially with cardiopulmonary disorders. Furthermore, the present system with capacitive coupling electrodes placed outside the bathtub wall appears to be superior to previous systems [16–18, 20] in terms of maintenance-free bathtub electrodes due to non-contact placement with tap water. It is also noted that the electrodes are completely hidden from the user’s field of view, unobtrusively measuring but leaving a user’s lifestyle otherwise unaffected.

As to medical applications, we had previously measured the ECG-RR intervals in hospital inpatients with cardiac failure using a bathtub system with conductive electrodes placed inside the bathtub wall [18], and the regression equation between the values of ECG/RR intervals obtained by the bathtub and the direct methods was *y(bathtub)* = 0.999*x(direct)* +0.843 with correlation coefficient *r* = 0.998, [17]. Through the clinical experiments, the artifacts due to body movements were also observed in these results and the abnormal rhythms could be certainly and frequently observed during bathing, though such arrhythmia could not be caught under normal medical examination. With this respect, the bathtub ECG measurement would be of clinical importance for a person to be cared for at home. Similarly, the measurement of respiratory rhythms would also be considered of value to obtain health conditions particularly in patients with heart failure [18].

Detection of drowning is hoped to provide better care for the elderly living alone at home. Drowning mortality (deaths per 100,000 population) was described in the World Health Organization Health Statistics and Information Services: Mortality data combined from 2009 to 2011 (updated at 13 November 2013) shows that the proportion of drownings involving a bathtub accident was the highest in Japan (13,634/21,108, 65%). In the 13,634 drowning deaths in Japan, the elderly (over 65 years old) was made up of a majority of 12,038 cases (88%) [29]. The highest rate of mortality in Japan is basically caused by habitual difference in bathing as compared to the other developed countries. Taking these circumstances into consideration, cardiopulmonary measurement during bathing can be fairly beneficial not only in the hospital but also at home.

To achieve this drowning detection using the present system, a considerably higher detection accuracy of arrest of breathing (approaching 100%) will be necessary. Although the amplitude of the respiration signal during simulated drowning decreased compared to those obtained from the other phases (see Fig. 8), the signal was somewhat fluctuated. This means that the detection accuracy of respiratory arrest is not yet fully adequate and thus further accurate detection of the respiratory signal is needed for this purpose. In contrast, artifactual signals due to a subject’s body motions would be helpful to indicate non-drowning periods, that is, the subject is active during bathing (he/she is alive), even though the bathtub system could not obtain ECG signals.

The present study is limited by several factors; first, all of the participants were young and healthy adults and it could be argued that the sample size was not large enough to conclusively evaluate the accuracy of the bathtub method. It is therefore recommended that the method should be re-evaluated across a wide age range of participants including patients with cardiopulmonary diseases.

Second, to detect more accurate ECG and respiratory signals, a detection circuit with high pass filter of lower cut off frequency is needed. The third driven electrode [27] or shielding all around the bathtub may also be useful to reject noise. Detection of the respiration curve including lower frequency components of the ECG signal [17] will be needed to improve measurement accuracy of respiratory intervals to allow drowning detection.

Third, in practical use during bathing, technical advancements to detect body movements such as posture changes, body washing actions, and so on, will be necessary. Although the present bathtub system allows measurement of ECG/RR and respiration intervals with reasonable accuracy as shown in Figs. 7 and 9, further investigation will be needed to obtain more stable ECG signals with less artifacts and noise. This could be technically addressed to develop an algorithm for detecting body motions using signal fluctuation analyses. We have also been contemplating investigating a method of canceling body notion artifacts with the use of a strain signal of the bathtub-flexure due to body movements.

Fourth, the detection of presence of person will be also needed for practical use during bathing. With respect to this, one study has already demonstrated detailed activity detection in the bathtub [30]. We have also been investigating the presence of a person in the bathtub to measure water height or a bathtub’s strain change due to the body weight. Furthermore, the presence of a person could also be recognized by detecting large artifacts superimposed on ECG signals when a subject is entering or exiting the bathtub.

The present prototype system with the capacitive coupling electrodes placed outside the bathtub wall allows ECG measurement with satisfactory accuracy, but further investigation will be necessary to improve on the following points; reducing the size and simplifying the electrode construction for much less noise detection of ECG signal and easier fixation of electrodes to the bathtub wall.

## Conclusion

This paper presents a new technique of cardiopulmonary measurement in a bath through tap water utilizing capacitive coupling electrodes placed outside the bathtub wall. It was demonstrated that the ECG signals could be successfully detected using this technique and that the ECG/RR and respiratory intervals derived from the ECG/RR intervals were determined with reasonable accuracy when compared to the conventional direct methods. Further technical improvements will be needed towards practical monitoring such as evaluation across a wide age range of participants including patients with cardiopulmonary diseases, redesign of filter circuit and electrode to obtain more accurate ECG and respiratory signals, cancel of body motion artifacts, and so on. Through these improvements, this method would be useful for the monitoring of a user’s health condition as part of his/her bathing routine without the need to interact with the device.

## Abbreviations

ECG: electrocardiogram
RR: peak-to-peak of R point of electrocardiogram
